# Left Atrial Compression by an Intramural Hematoma: A Rare Case of Obstructive Shock

**DOI:** 10.7759/cureus.66049

**Published:** 2024-08-03

**Authors:** Georgios Dividis, Dimitrios Pallas, Ioannis Karageorgiou, Foteini Lazaridou

**Affiliations:** 1 Department of Cardiology, Agios Pavlos General Hospital of Thessaloniki, Thessaloniki, GRC; 2 Department of Internal Medicine, Agios Pavlos General Hospital of Thessaloniki, Thessaloniki, GRC

**Keywords:** transthoracic echocardiography, rare case of shock, transthoracic echocardiogram, intramural hematoma type b, left atrium compression, obstructive shock

## Abstract

A 3.5 cm diameter descending aorta focal aneurysm was incidentally found when a computed tomography (CT) was conducted due to persistent pyrexia in an 85-year-old woman hospitalized for a non-obstructive urinary tract infection. Ten days later, whilst fever subsided and inflammation markers decreased, she became hypoxic. CT revealed an aortic intramural hematoma (Stanford type B) increasing the diameter of the thoracic aorta aneurysm to 6.5 cm. A thoracic endovascular aortic repair (TEVAR) surgery was performed. Seven days after the operation she developed respiratory and hemodynamic compromise. CT depicted further enlargement of the aortic intramural hematoma, increasing the aortic diameter to 8 cm. Transthoracic echocardiography provided valuable information showing extrinsic compression of the left atrium and left ventricle inflow obstruction provoking obstructive shock.

## Introduction

Obstructive shock is one of the least common causes of shock, with septic, cardiogenic, and hypovolemic shock being the most prevalent forms in everyday clinical practice [1]. In cases of pericardial effusion, tension pneumothorax, vena cava syndrome, mediastinal tumors, and ventilation with high positive end-expiratory pressure (PEEP) level, obstructive shock is caused by an impaired diastolic filling and a reduced cardiac preload [2]. Conversely, pulmonary embolism and space-occupying masses in the mediastinum elevate the afterload on the right ventricle while simultaneously reducing the preload on the left ventricle [2].

Although transthoracic echocardiography (TTE) is not the examination of first choice in such situations, it can be of help in diagnosis as it is easily applicable and can provide significant information for a patient’s hemodynamic status, even in critical conditions [3,4]. In our case, an intramural hematoma (IMH) of the descending thoracic aorta acted like a mediastinal space-occupying mass creating circumstances of obstructive shock. Herein, we describe a rare case of obstructive shock attributable to extrinsic compression of the left heart cavities in an elderly patient with an aneurysm of the thoracic aorta. 

## Case presentation

An 85-year-old woman presented to the emergency department with fever with chills starting four days ago. She also complained of concomitant dysuria. Her past medical history included arterial hypertension, macrocytic anemia, depression, hysterectomy due to cervical cancer, and cholecystectomy. Her outpatient medical treatment included bisoprolol, manidipine, B12 supplementation intramuscularly, alprazolam, and sertraline. 

On presentation, blood pressure was 155/60 mmHg, heart rate was 70 bpm, oxygen saturation was 98% on ambient air, and temperature was 38.2^o^C. Clinical examination of the abdomen and lungs showed no abnormal findings, cardiac auscultation revealed no cardiac murmurs. Full blood count demonstrated macrocytic anemia, leukocytosis with neutrophilia, and elevated platelet count. Further biochemical testing revealed elevated levels of C-reactive protein (CRP), procalcitonin (PCT), urea, and creatinine, indicating an inflammatory reaction and acute kidney injury (AKI) (Table 1). Upper abdomen and kidney-ureter-bladder ultrasound were unrevealing. Considering the patient’s presenting complaints, the above results indicated a non-obstructive urinary tract infection complicated by AKI.

**Table 1:** Laboratory test results at admission ESR: erythrocyte sedimentation rate; SGOT: serum glutamic oxaloacetic transaminase; SGPT: serum glutamic pyruvic transaminase

Parameters	Values	Reference range
White Blood Cells (cells/μL)	13.570	4.500 - 11.000
Polymorphonuclear neutrophils (cells/μL)	11.032	1.500 - 8.000
Hemoglobin (g/dl)	9.5	12 - 16
Hematocrit (%)	27.6	36 - 48
Mean Corpuscular Volume (fl)	103	80 - 100
Platelets (cells/μL)	610.000	150.000 - 350.000
International Normalized Ratio	1.38	<1.20
Urea (mg/dl)	67	5 - 40
Creatinine (mg/dl)	1.74	0.5 - 1.3
Glucose (mg/dl)	117	70 - 100
Sodium (mmol/l)	139	135 - 145
Potassium (mmol/l)	4.2	3.5 - 5.0
SGOT (IU/ml)	17	8 - 40
SGPT IU/ml)	15	8 - 55
C-Reactive Protein (mg/dl)	30.9	<0.5
Procalcitonin (ng/ml)	0.92	<0.05
Lactose Dehydrogenase (IU/L)	147	140 - 280
Creatine Phosphokinase (μg/L)	27	10 - 120
ESR (mm/h)	120	<30

Urine and blood samples were sent for cultures. Intravenous fluids and empiric antibiotic treatment with ciprofloxacin were initiated. Both urine and blood cultures grew *Escherichia coli* sensitive to the antibiotic that was empirically chosen. Interestingly, despite normalization of renal function and improvement of inflammatory markers, pyrexia persisted.

**Figure 1:**
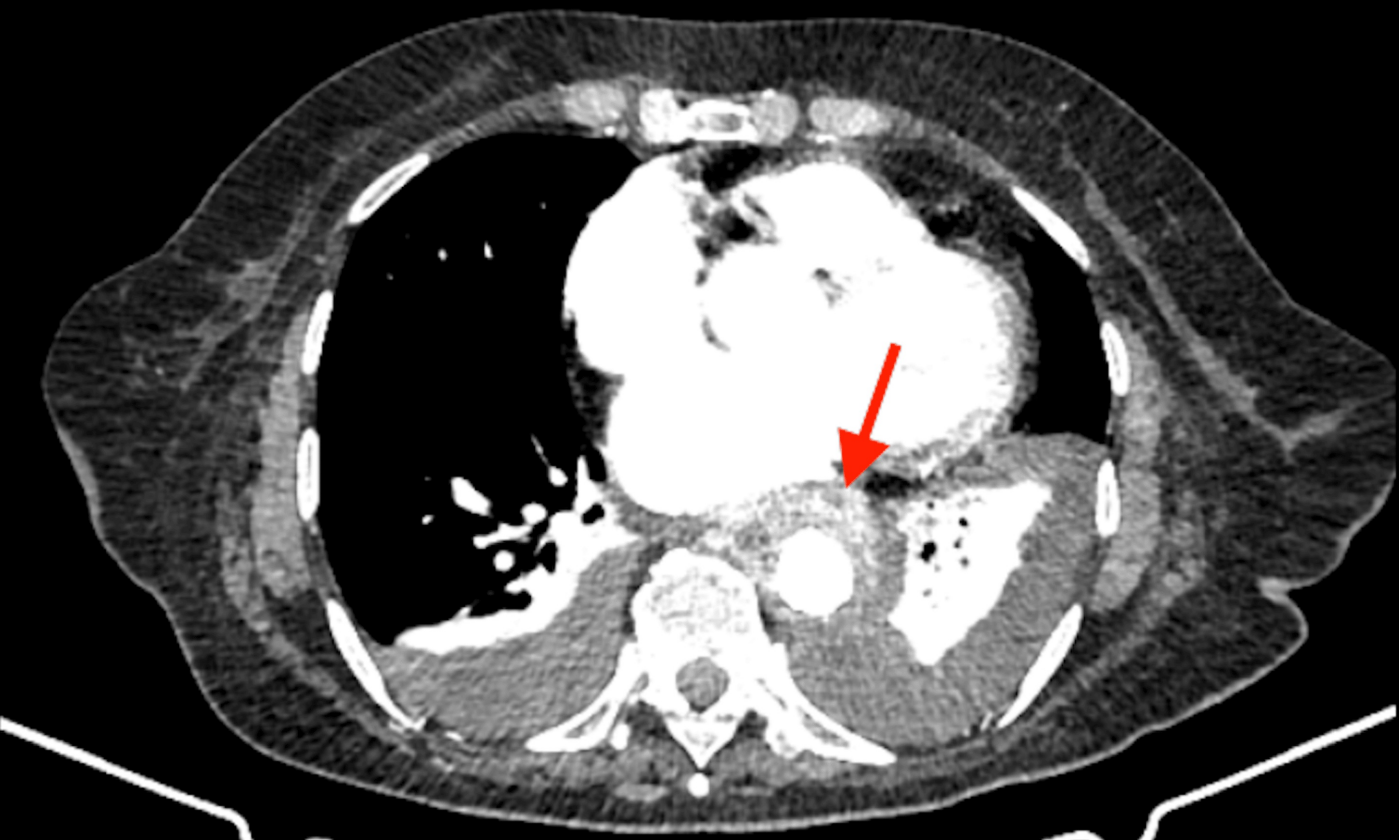
Computed tomography depiction of focal aortic aneurysm of the descending aorta (arrow).

**Figure 2:**
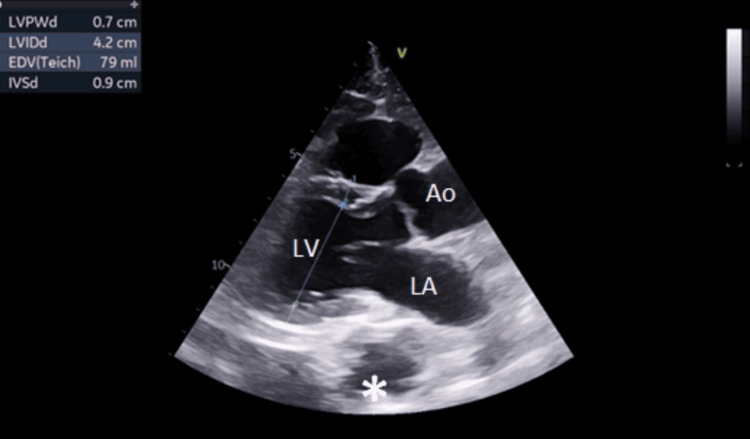
Normal size, function, and contractility of the left ventricle. Focal aneurysm of the descending thoracic aorta (asterisk) can be seen, causing no hemodynamic changes. Left atrium, left ventricle, and ascending aorta are depicted. LV: left ventricle; LA: left atrial; Ao: ascending aorta

**Figure 3:**
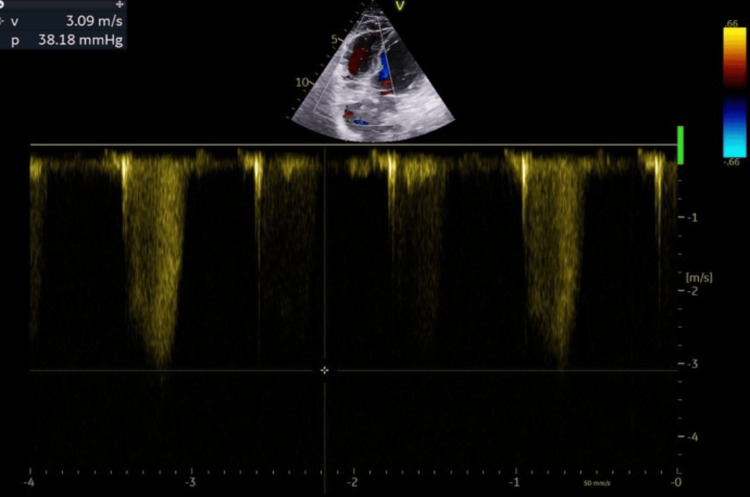
Μild pulmonary hypertension Triscupid regurgitation velocity (>2,5 m/s) indicating mild pulmonary hypertension

**Figure 4:**
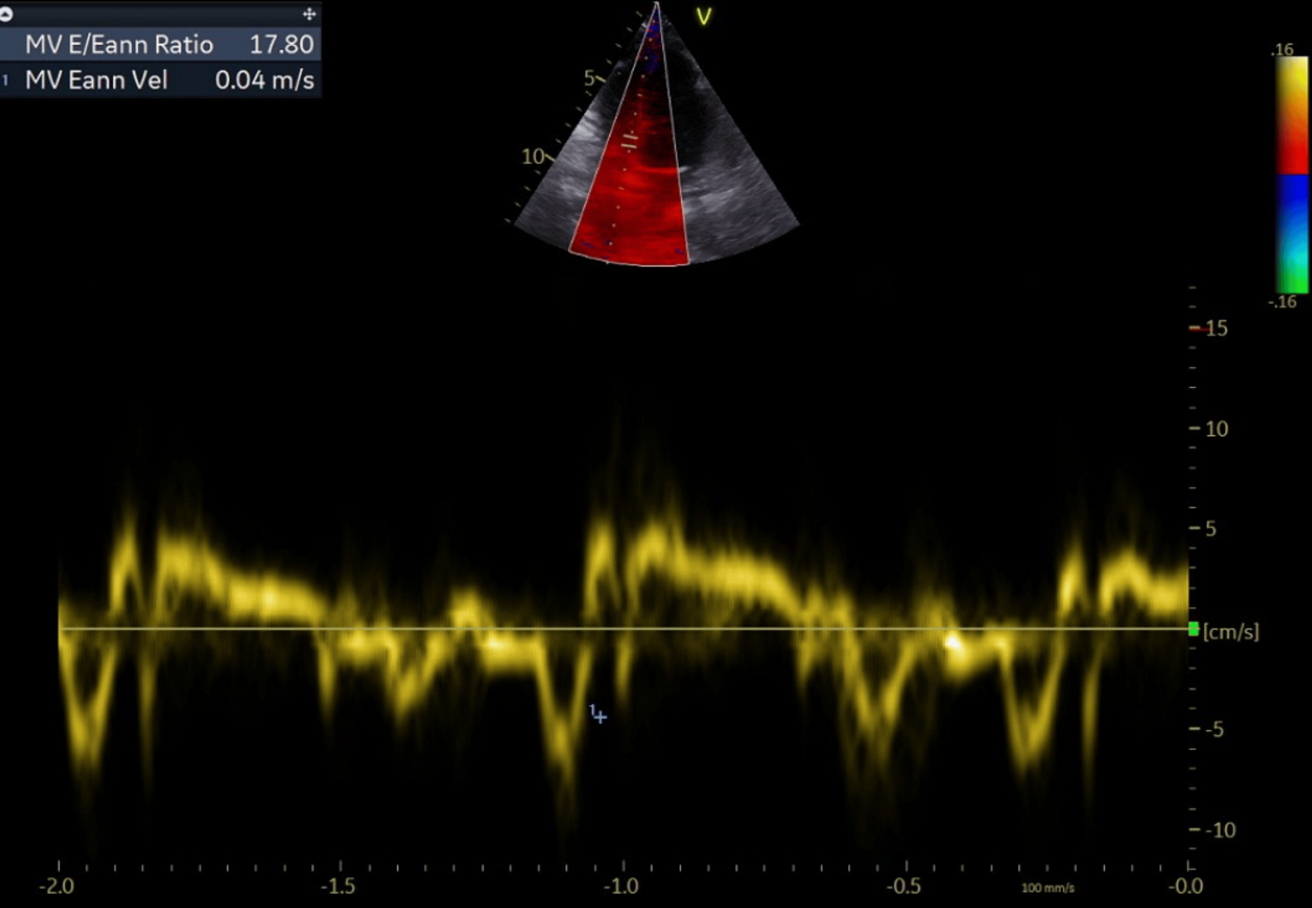
Evaluation of diastolic dysfunction (E/E’=17) The E wave is the early diastolic filling wave seen on Doppler interrogation of the mitral valve. The E' wave is acquired in tissue Doppler either of the basal or the lateral wall (in our case we used the mean value) of the mitral annulus. Values greater than 15 suggest that left ventricle end-diastolic pressure is elevated.

A computed tomography (CT) of the abdomen and pelvis was performed, without revealing an abdominal or renal abscess or any other inflammatory source. A 3.5 cm diameter focal aneurysm of the descending aorta was incidentally found (Figure 1). A transthoracic echocardiography (TTE) confirmed the presence of a focal aneurysm of the descending aorta, in the parasternal long-axis view confirming the CT findings (Figure 2). Additionally, it revealed an enlargement of both atriums, mild pulmonary hypertension, and an elevated left ventricular end-diastolic pressure (Figure 3, 4). After vascular surgery consultation, a conservative approach was recommended. The fever resolved after completing a seven-day antibiotic regime.

**Figure 5:**
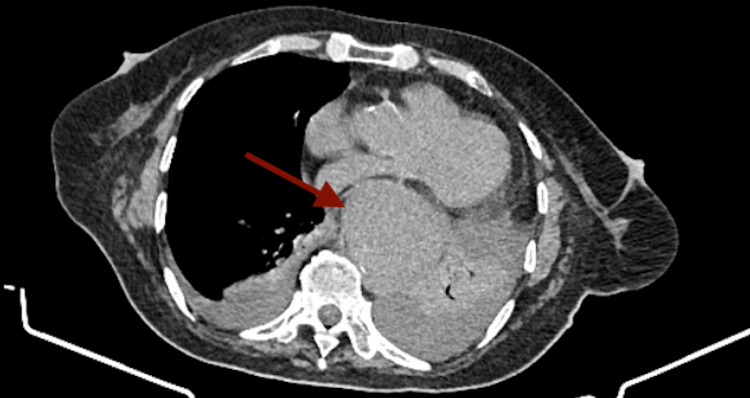
Computed tomography depiction of aortic intramural hematoma (arrow) increasing the diameter of thoracic aorta to 6.5 cm.

Three days after the fever resolved, she became hypoxic with an oxygen saturation of 89% on ambient air. Chest X-ray revealed bilateral pleural effusion with a predominance on the left. Diagnostic thoracentesis showed transudative pleural effusion according to Light’s criteria. Furosemide was initiated and oxygenation was supported via nasal cannula at 3 lt/minute. For further evaluation, chest CT angiography (CTA) was performed which interestingly revealed a descending aorta IMH, Stanford type B, increasing the diameter of the aneurysm to 6.5 cm (Figure 5) [5]. The patient was transferred to the vascular surgery department and thoracic endovascular aortic repair (TEVAR) intervention was performed. 

**Figure 6:**
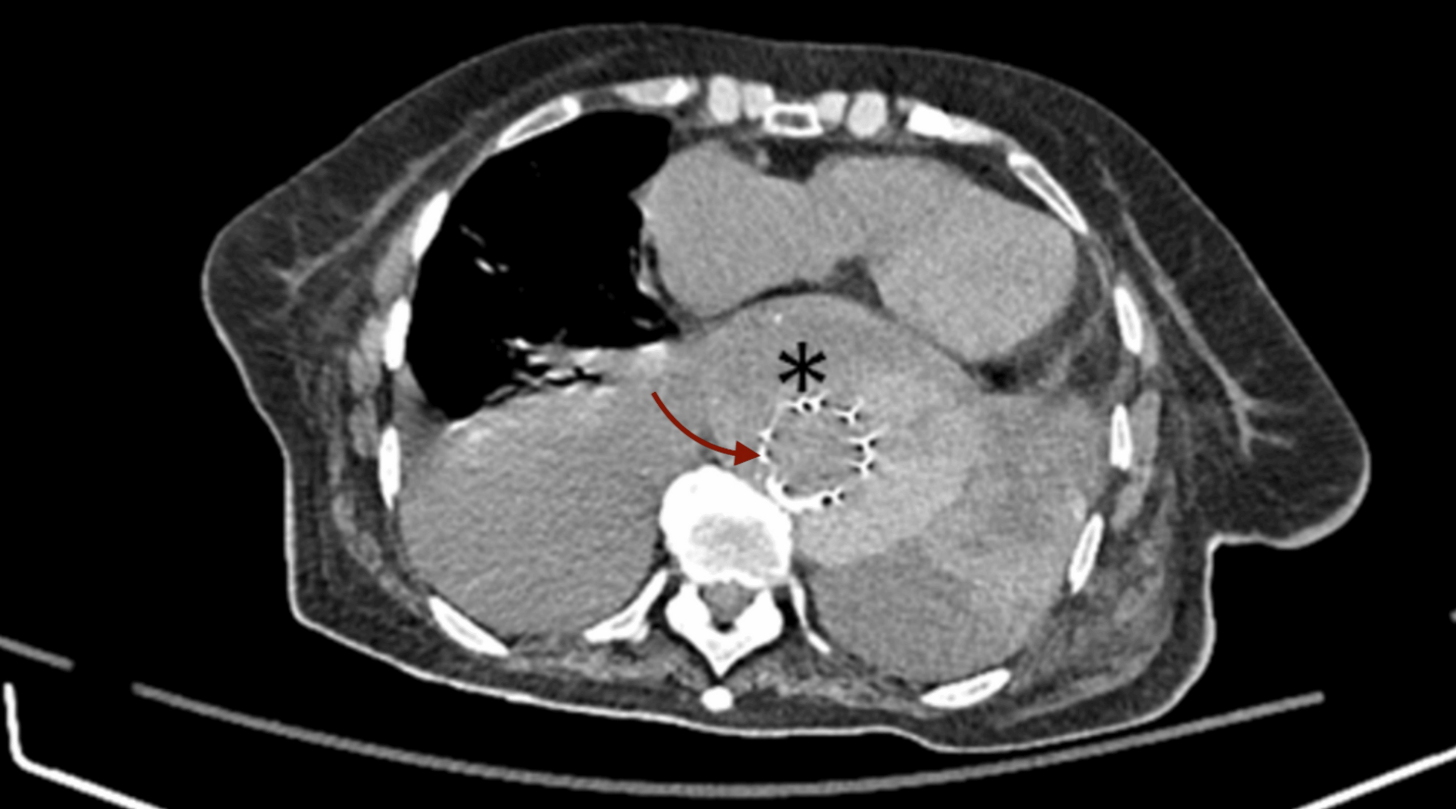
Further enlargement of the aortic intramural hematoma (asterisk), increasing the aortic diameter to 8 cm. Large bilateral pleural effusion. Arrow indicates the stent after thoracic endovascular aortic aneurysm repair (TEVAR)

**Figure 7:**
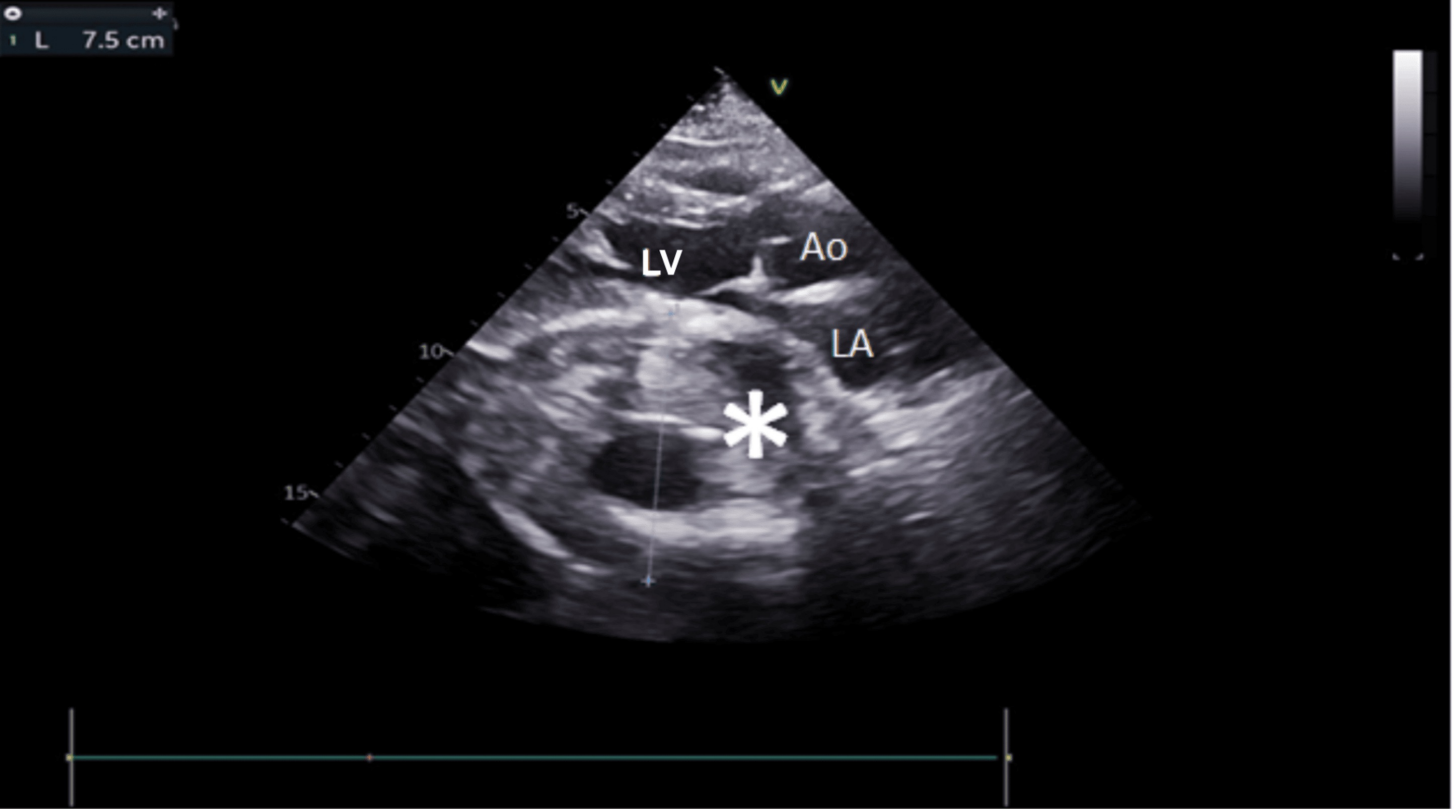
Parasternal long-axis view showing extrinsic compression of the left atrium by a type B aortic aneurysm IMH (asterisk). A left ventricle inflow obstruction is observed. LA: left atrium; LV: left ventricle; Ao: ascending aorta

**Figure 8:**
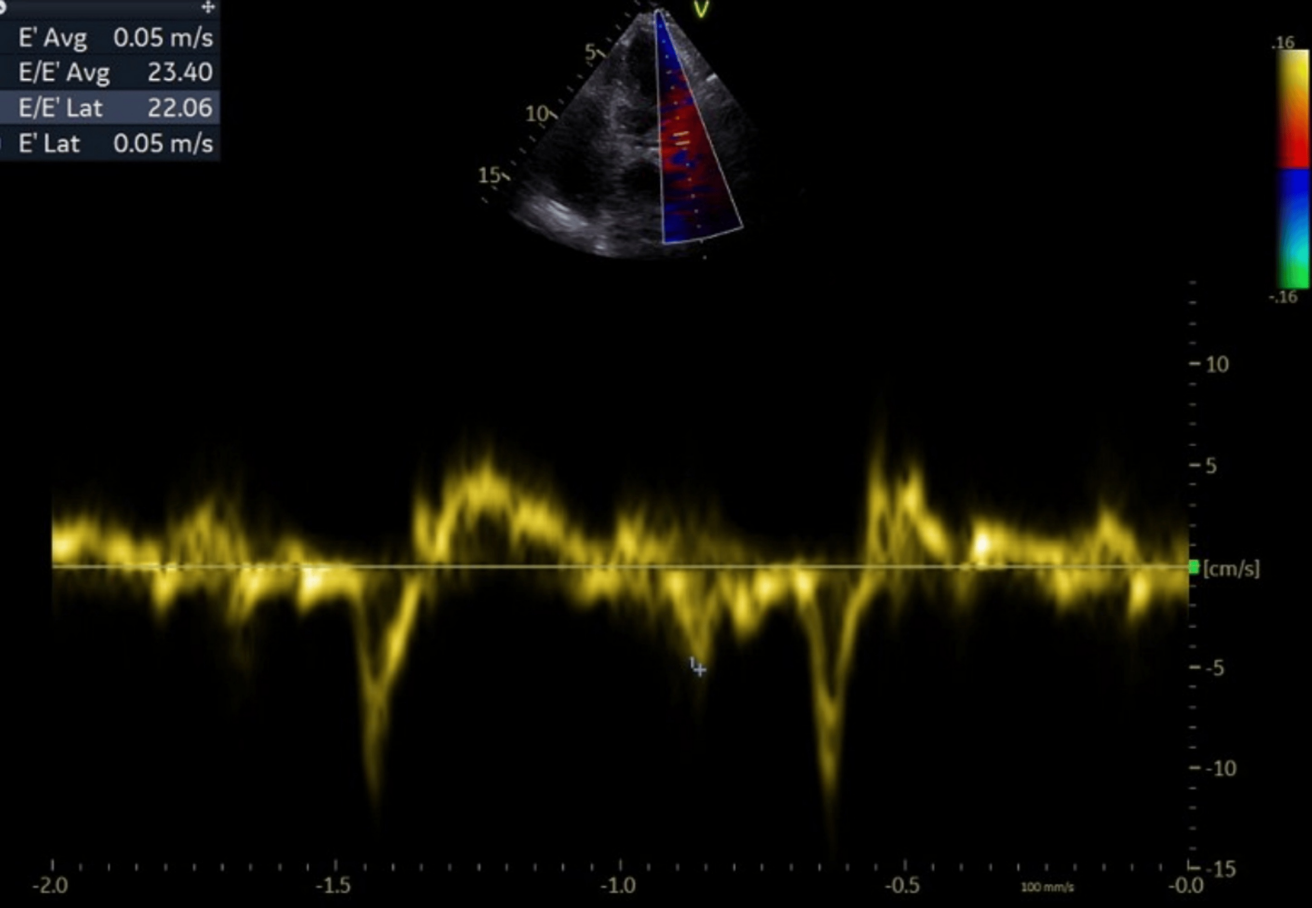
Evidence of elevated end-diastolic pressure of the left ventricle (E/E’ avg=23.4). A further increase of this ratio indicates a deterioration of the diastolic dysfunction. The E wave is the early diastolic filling wave seen on Doppler interrogation of the mitral valve. The E' wave is acquired in tissue Doppler either of the basal or the lateral wall (in our case we used the mean value) of the mitral annulus. Values greater than 15 suggest that left ventricle end diastolic pressure is elevated.

**Figure 9:**
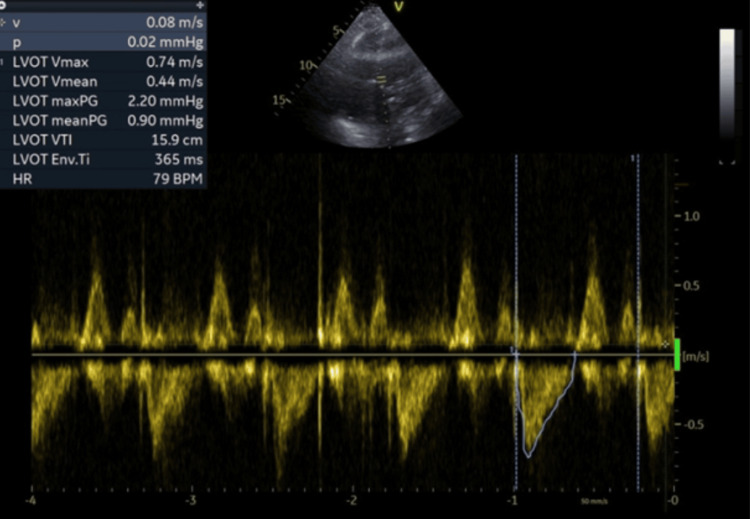
Measurement of velocity time integral (VTI) left ventricle outflow tract (LVOT)

Seven days after the intervention, her cardiorespiratory status deteriorated as manifested by worsening dyspnea, hypoxia, hypotension, and tachycardia. Fine crackles were identified on lung auscultation. A plain chest X-ray revealed pulmonary edema. Electrocardiogram revealed atrial fibrillation with fast ventricular response which was pharmaceutically cardioverted using amiodarone intravenously. Emergency CTA depicted a further enlargement of the aortic IMH, increasing the aortic diameter to 8 cm and large bilateral pleural effusion (Figure 6). An increase of the pleural effusion was also noticed. Bedside TTE revealed an echogenic circular mass extrinsically compressing the left atrium with a major diameter of 80 mm. A crescent-shaped left atrial cavity remained while the aforementioned hematoma of the descending aorta was creating a left ventricle inflow obstruction (Figure 7). Left ventricular wall motion and ejection fraction were normal. Measurement of the left ventricle outflow tract area and its velocity time integral confirmed low cardiac output (Figures 8, 9) (Table 2). Therefore, hemodynamic compromise was attributed to low cardiac output due to low preload induced by the IMH compression of the left atrium. Hypoxia was attributed to pulmonary edema caused by backward transmission of left atrium elevated pressure to the pulmonary veins. Septic etiology was not considered probable due to the absence of pyrexia and elevated inflammatory markers. 

**Table 2:** Measurement of cardiac output LVOT diameter: 19 mm; LVOT VTI: 15.9 cm; Heart rate: 79 bpm; Weight: 65 kg; Height: 1.55 m Cardiac output (CO)= SV X HR Cardiac index (CI) = CO/BSA Stroke volume (SV) = LVOT area x LVOT VTI Stroke volume index (SVi) = SV/BSA BSA: body surface area; LVOT: left ventricle outflow tract; VTI: velocity time integral; HR: heart rate

Variables	Values
Cardiac output	3.6 L/min; N.R. 4-8
Cardiac Index	2.13 L/min/m^2^; N.R. (2.5-4)
Stroke volume	45 mL/beat; N.R (60-100)
Stroke volume index	27 Ml/beat/m^2^; N.R (33-47)

Intravenous vasoconstrictor administration was initiated, treatment with bisoprolol was interrupted, and diuresis was intensified. Oxygen supplementation was escalated to a high-flow nasal cannula at 60 lt/minute and 90% fraction of inspired oxygen (FiO2). Open surgical repair of the aortic aneurysm, evacuative thoracentesis, or further escalation of care was not applied due to her poor performance status. Her cardiorespiratory status gradually deteriorated and she passed away after 30 days of hospitalization. 

## Discussion

The left atrium is a thin wall chamber of the heart with low intracavitary pressure connecting the lungs with the left ventricle. It is located inferoposteriorly compared to the other chambers of the heart and drives the blood from pulmonary veins to the left ventricle via the mitral valve. Its thin wall and low pressure make it vulnerable to impressions from nearby structures. According to van Rooijen et al., there are four categories of structures that can cause encroachment or compression of the left atrium: gastrointestinal tract, mediastinum structures, aorta/intrapericardial structures, and lung structures (Table 3) [6]. 

**Table 3:** Origin of the causes of extrinsic encroachment or compression of the left atrium

Structures	Causes
Gastrointestinal tract	Diaphragmatic and esophageal hernia
	Esophageal leiomyosarcoma
	Achalasia
	Chronic gastric volvulus by a para-esophageal hernia and a diaphragmatic hernia
Mediastinum	Mediastinal lymphoma
	Mediastinal schwannoma
	Sarcoidosis
	Thymoma
Aorta/intrapericardial structures	Ascending thoracic aortic aneurysm
	Descending thoracic aortic aneurysm
	Aortic root dilatation and scoliosis
	Pseudoaneurysm with subepicardial dissection onto the left atrial wall
	Hematoma from rupture of type B aortic dissection
	Pericardial cyst
	Pericardial hematoma
Pulmonary structures	Lung tumor
	Bronchogenic cyst

Left atrial impression has been subdivided by D’Cruz et al. according to the severity of anatomical deformation and its hemodynamic impact [3]. Three different classes have been formed: (i) proximity (by an adjacent structure without chamber deformation), (ii) encroachment (distortion of normal cardiovascular architecture without hemodynamic effect), and (iii) compression (where impression leads to severe inflow obstruction causing hemodynamic instability). Compression status is the condition always leading to symptoms while the other two may remain clinically silent.

Acute aortic syndromes are emergency conditions involving the aorta. IMH, penetrating atherosclerotic ulcer, and aortic dissection are conditions sharing as common features a breach in the integrity of the aortic wall. In IMH, blood, leaking from vasa vasorum, ruptures into the aortic media tunica at low pressure, forming a thrombus pushing the outer wall of the aorta outward while the aortic lumen remains normal [5]. Progression to aortic dissection due to rupture of the intima is a condition that may complicate IMH. In our case, initially, an uncomplicated type B IMH developed, and a conservative approach was preferred (Table 4) [5]. The conservative approach was revisited due to the appearance of poor prognostic factors predisposing to rupture such as maximum aortic diameter of more than 50 mm, progressive maximum aortic wall thickness of more than 11 mm, and recurrent pleural effusion (Table 5). Considering the above circumstances, TEVAR was performed. 

**Table 4:** Conservative approach for aortic diseases according to ESC clinical practice guidelines ESC: European Society of Cardiology; IMH: intramural hematoma; TEVAR: thoracic endovascular aortic repair

Recommendations on the management of intramural hematoma	
In cases of type B IMH, initial medical therapy under careful surveillance is recommended	I C
In complicated type B IMH, TEVAR should be considered	IIa C

**Table 5:** Poor prognostic factors as part of the ESC guidelines describing the natural history, morphologic changes, and complications of IMH IMH: intramural hematoma; ESC: European Society of Cardiology

Predictors of intramural hematoma complications
Persistent and recurrent pain despite aggressive medical treatment
Difficult blood pressure control
Ascending aortic involvement
Maximum aortic diameter >50 mm
Progressive maximum aortic wall thickness (>11 mm)
Enlarging aortic diameter
Recurrent pleural effusion
Penetrating ulcer or ulcer-like projection secondary to localized dissections in the involved segment
Detection of organ ischemia (brain, myocardium, bowels, kidneys)

The diagnostic value of TTE for extra-cardiac structures is low and inferior to CT [7]. CT and magnetic resonance imaging (MRI) are the leading techniques for the diagnosis and classification of IMH [5]. The sensitivity of TTE for the detection of IMH is estimated to be lower than 40%. However, TTE provides several advantages as it can readily be performed at the patient’s bedside, in any hospital, without the need to transfer the hemodynamically unstable patient. Additionally, echocardiography is recommended as the modality of first choice for the diagnosis of shock [8]. Taking into consideration all of the above, the combination of CT and TTE is estimated to be the best imaging strategy for the diagnosis of IMH and its complications [9].

Our patient presented with an unusual cause of both circulatory shock and pulmonary edema. The compression of IMH to the left atrium caused left ventricular inflow obstruction and, as a result, a reduction in the preload of the left ventricle. This in turn led to low cardiac output, as documented by TTE. Additionally, the compression induced an elevation of left atrium pressure and, subsequently, of the pulmonary capillary wedge pressure (PCWP) [10], leading to pulmonary edema. Left ventricle systolic function was preserved while a further elevated LVEDP was observed. These findings prove that there was no intrinsically abnormal myocardial function. In our case, the IMH appeared as a mediastinal space-occupying mass that compressed the left atrium and pulmonary veins provoking obstructive shock [2]. 

## Conclusions

We diagnosed a rare and unique cause of obstructive shock due to extrinsic compression of the left atrium by an IMH type B. This case highlights the role of TTE in the diagnosis and assessment of IMH and its complications. 
